# Larval Starvation to Satiation: Influence of Nutrient Regime on the Success of *Acanthaster planci*

**DOI:** 10.1371/journal.pone.0122010

**Published:** 2015-03-19

**Authors:** Kennedy Wolfe, Alexia Graba-Landry, Symon A. Dworjanyn, Maria Byrne

**Affiliations:** 1 School of Medical Sciences, The University of Sydney, Sydney, NSW 2006, Australia; 2 National Marine Science Centre, Southern Cross University, Coffs Harbour, NSW 2450, Australia; 3 School of Biological Sciences, The University of Sydney, Sydney, NSW 2006, Australia; Leibniz Center for Tropical Marine Ecology, GERMANY

## Abstract

High density populations of the crown-of-thorns seastar, *Acanthaster planci*, are a major contributor to the decline of coral reefs, however the causes behind periodic outbreaks of this species are not understood. The *enhanced nutrients hypothesis* posits that pulses of enhanced larval food in eutrophic waters facilitate metamorphic success with a flow-on effect for population growth. The *larval resilience hypothesis* suggests that *A*. *planci* larvae naturally thrive in tropical oligotrophic waters. Both hypotheses remain to be tested empirically. We raised *A*. *planci* larvae in a range of food regimes from starvation (no food) to satiation (excess food). Algal cell concentration and chlorophyll levels were used to reflect phytoplankton conditions in nature for oligotrophic waters (0-100 cells ml^-1^; 0-0.01 μg chl *a* L^-1^), natural background levels of nutrients on the Great Barrier Reef (GBR) (1,000-10,000 cells ml^-1^; 0.1-1.0 μg chl *a* L^-1^), and enhanced eutrophic conditions following runoff events (100,000 cells ml^-1^; 10 μg chl *a* L^-1^). We determine how these food levels affected larval growth and survival, and the metamorphic link between larval experience and juvenile quality (size) in experiments where food ration per larvae was carefully controlled. Phytoplankton levels of 1 μg chl *a* L^-1^, close to background levels for some reefs on the GBR and following flood events, were optimal for larval success. Development was less successful above and below this food treatment. Enhanced larval performance at 1 μg chl *a* L^-1^ provides empirical support for the *enhanced nutrients hypothesis*, but up to a limit, and emphasizes the need for appropriate mitigation strategies to reduce eutrophication and the consequent risk of *A*. *planci* outbreaks.

## Introduction

Coral reef ecosystems are in global decline due to anthropogenic impacts such as overfishing, increased pollution, sedimentation and disease, and global change stressors such as ocean warming and acidification [1–3]. The coral-eating crown-of-thorns seastar, *Acanthaster planci*, is a major contributor to the decline of coral reefs [4,5]. Over recent decades, hard coral cover on the Great Barrier Reef (GBR) has decreased by around 0.53% yr^-1^, with an estimated 42% of this negative growth attributed to predation by *A*. *planci* outbreak populations [4,6,7]. However, these predicted values are suggested to overestimate the total impact of this seastar because they are often based on selective, small-scale studies [8].

Outbreaks, the periodic increase in population density of *A*. *planci*, are a global issue on reefs from the Red Sea to the Indo-Pacific [9–12], although chronic (+30 yr) high-density populations also occur [13]. For the GBR, modelling indicates that outbreak populations arise from the Cooktown-Cairns area, with downstream connectivity patterns generating secondary outbreaks [14,15]. Historically, outbreaks of *A*. *planci* suggested to have increased from around one outbreak every 50–80 years to one every 15 years on the GBR [14] and elsewhere [11]. At this rate, coral reefs may be unable to recover [4,6,16], especially as calcifying organisms like corals are highly vulnerable to the stressors associated with global change [3,17].

The ecological drivers of *A*. *planci* outbreaks have been a subject of much interest and controversy for decades, with several hypotheses proposed [12,18]. One hypothesis suggests that *A*. *planci* outbreaks are a natural phenomenon that have occurred through the evolutionary history of this species, reflecting the boom and bust life history characteristic of opportunistic echinoderm species [9,19]. It is also suggested that periodic increases in *A*. *planci* populations are important in enhancing coral species diversity on coral reefs, as an intermediate disturbance [20]. The ability of the planktotrophic larvae of *A*. *planci* to develop in oligotrophic tropical waters indicates that they are tolerant of low phytoplankton levels and that they may be able to use other nutrient sources (e.g. dissolved organic matter—DOM)—the *larval resilience hypothesis*—a feature that may facilitate the success of this species [21,22].

Several hypotheses attribute anthropogenic activities to outbreaks of *A*. *planci* [12]. The *predator removal hypothesis* suggests that populations are largely controlled by predation, and that overfishing may facilitate increased survival of *A*. *planci* to maturity [10,23]. There is a greater vulnerability of fished zones to *A*. *planci* outbreaks compared with no-take zones due to decreased predation pressure [24,25]. Since the early juvenile stage is a potential population bottleneck where mortality is predicted to be ~99% during the first year [26], predation of juvenile *A*. *planci* by small benthic predators may also be important [27,28]. The striking chemically and structurally defended body of *A*. *planci*, even at the juvenile stage, suggests an evolutionary design for protection against predators.

*Acanthaster planci* has a high-risk, high-gain life history, and outbreaks are suggested to be due to pulses of larval success [19]. This seastar is highly fecund with a particularly robust fertilisation biology [29]. Thus even small increases in survival during the planktonic and juvenile stages could have significant flow-on effects for adult success [27,30]. However, other tropical and sympatric asteroid species with a similar planktotrophic life history do not exhibit marked population fluctuations, and so the opportunistic nature of the larvae of *A*. *planci* remains a challenge to understand.

Enhancement of the phytoplankton food of *A*. *planci* larvae due to coastal eutrophication is suggested to have altered the population dynamics of this species [31]. The *enhanced nutrients hypothesis* posits that increased phytoplankton for larval *A*. *planci* due to eutrophication from river runoff, especially linked to storms and floods, is the driving factor behind modern outbreaks [28,30,31]. Increased chlorophyll *a* (chl *a*) concentrations—a proxy for phytoplankton abundance—occur on the GBR following major storms, especially during the wet season when *A*. *planci* larvae are in the plankton [32–35]. However, since outbreaks on the GBR are more prevalent on reefs at a distance from major river systems [12], the links between runoff and larval population dynamics are tenuous. Furthermore, naturally high phytoplankton levels in other tropical regions are not necessarily associated with *A*. *planci* outbreaks, suggesting that high nutrient levels and enhanced larval food may not be the driver of outbreaks of this seastar [36].

Although the *enhanced nutrients hypothesis* has received considerable traction [14,28,37], there remains a lack of empirical data on larval responses to food regimes, especially in an ecologically robust context with respect to the algal and chl *a* concentrations experienced by the larvae in nature [12]. As a tropical species adapted to life in oligotrophic waters across its Indo-Pacific distribution, it is important to understand the responses of the larvae to conditions typical of these reef systems. Although the development of *A*. *planci* is well described [14,21,30,37], inferences on how larval performance in these studies applies to nature is not clear since chl *a* values were extrapolated from published values of chl *a* in different phytoplankton species [30]. Moreover, since these studies did not adjust for the influence of larval mortality and thus changing larval density on food availability per individual, it is not possible to discern between absolute food levels in treatments, and whether larvae were starved or overfed [14,37]. Thus, it is difficult to apply the results of previous studies to the predictions of the *enhanced nutrients hypothesis*.

As one of the most important species influencing the integrity of Indo-Pacific coral reef ecosystems, it is critical to understand the drivers of *A*. *planci* outbreaks so that effective management strategies can be devised and implemented. In this study, we focused on the larval stage and its response to different food levels to determine the effects of food concentration on larval development and recruitment success of *A*. *planci*. We reared larvae in algal cell densities simulating starvation/oligotrophic waters, natural background levels of nutrients on the GBR, and enhanced eutrophic conditions in runoff scenarios. Our experiments were placed in context with chl *a* data determined for the GBR coinciding with the time when *A*. *planci* larvae are in the plankton (eReefs: http://www.bom.gov.au/marinewaterquality/). We monitored algal cell densities and chl *a* levels as a proxy for natural phytoplankton. Rearing conditions were carefully monitored to maintain a consistent larval:algal cell ratio in each treatment through to the juvenile settlement stage.

We predicted that as a species adapted to tropical oligotrophic waters, the larvae of *A*. *planci* would be resilient to low nutrient levels, and would develop successfully in low food treatments. As an ecologically opportunistic species that can quickly benefit from increased food supply (e.g. runoff conditions), high nutrient levels were expected to be beneficial to larval growth and support higher survival to the juvenile stage, thereby providing empirical data to support the *enhanced nutrients hypothesis*.

## Methods

### Levels of chlorophyll a on the Great Barrier Reef

**Fig 1:**
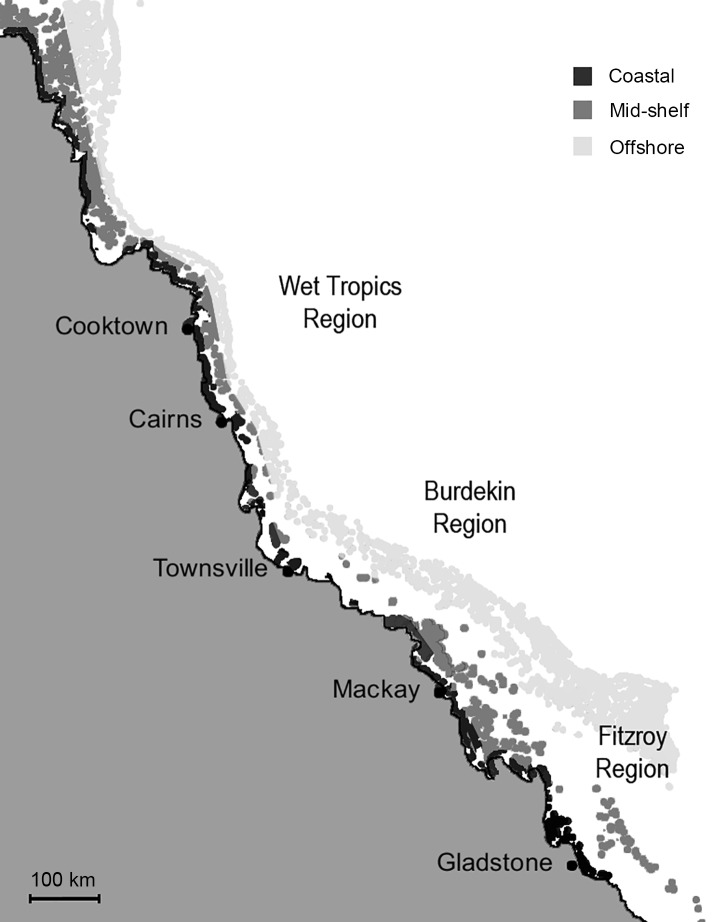
Regions on the Great Barrier Reef where hotspots of *Acanthaster planci* outbreaks occur (Wet Tropics, Burdekin, Fitzroy), with indication of coastal, mid-shelf and offshore reefs. The ‘initiation box’ for *A*. *planci* outbreaks between Cooktown and Cairns [12] is indicated by the rectangle.

With respect to natural levels of chl *a* on the GBR, satellite data collected by eReefs (http://www.bom.gov.au/marinewaterquality/) is congruent to that recorded in previous studies based on analyses by discrete water samples (S1 Table) [38,39]. Natural levels of chl *a* on the GBR were determined from reference data from eReefs for the time *Acanthaster planci* larvae would be expected in the plankton (five months; November-March). Monthly data, as provided by eReefs, were assimilated for four years (2010–2014) (n = 20; Table 1). Average natural chl *a* concentration (μg chl *a* L^-1^) was determined for regions on the GBR where hotspots of *A*. *planci* outbreaks occur; the Wet Tropics (Cairns/Lizard Island), Burdekin (Townsville) and Fitzroy (Swains Reef) Regions (Fig. 1; Table 1). In each region, data were divided into coastal, mid-shelf and offshore locations (Fig. 1).

**Table 1:** Average chl *a* concentration (μg L^-1^) where hotspots of *Acanthaster planci* outbreaks occur; Wet Tropics (Cairns/Lizard Island), Burdekin (Townsville) and Fitzroy (Swains Reef), during the time when larvae would be expected in the plankton (November-March, 2010–2014; n = 20; ±se).

		Chl *a* concentration		
Condition	Location	*Coastal*	*Mid-shelf*	*Offshore*
**Natural mean chl *a***	*Wet Tropics*	1.03 (±0.07)	0.48 (±0.04)	0.20 (±0.01)
	*Burdekin*	1.02 (±0.09)	0.35 (±0.04)	0.19 (±0.01)
	*Fitzroy*	1.12 (±0.13)	0.43 (±0.03)	0.22 (±0.01)
**Event mean chl *a***	*Wet Tropics*	1.16 (±0.16)	0.60 (±0.09)	0.23 (±0.02)
	*Burdekin*	1.37 (±0.14)	0.41 (±0.04)	0.20 (±0.01)
	*Fitzroy*	1.55 (±0.33)	0.57 (±0.10)	0.24 (±0.02)
**Event maximum chl *a***	*Wet Tropics*	10.75 (±0.98)	7.97 (±1.18)	3.41 (±0.91)
	*Burdekin*	10.64 (±1.10)	6.37 (±1.34)	1.66 (±0.23)
	*Fitzroy*	12.29 (±2.01)	6.72 (±1.78)	3.88 (±0.67)

The chl *a* data are presented as natural background levels not directly associated with a storm event, and levels determined following events (e.g. major floods, cyclones). Event mean and maximum chl *a* values were calculated for the week following a major cyclone or flood event in these locations (n = 7; ±se). All data was sourced from eReefs (http://www.bom.gov.au/marinewaterquality/).

In addition, mean and maximum chl *a* values were calculated using chl *a* values provided by eReefs for the week following major cyclone or flood events in these locations. These values were used to indicate pulses of enhanced chl *a* on the GBR when *A*. *planci* larvae are in the plankton. A total of seven storm and/or cyclone events (hereafter called events) were identified between 2009–2014 (Table 1).

### Specimen collection, spawning and rearing

Adult *A*. *planci* were collected on SCUBA in early November from the Great Barrier Reef near Cairns (16°55S, 145°46E), Australia (permitted by the Great Barrier Reef Marine Park Authority; permit number G13/36068.1). Individuals were packaged with oxygen-rich seawater and transported by air to Coffs Harbour, Australia. The seastars were acclimated in aquaria with flow-through seawater at the National Marine Science Centre, Southern Cross University, for 1–2 weeks at ambient temperature for the collection habitat during the time of collection (~27°C; eReefs: http://www.bom.gov.au/marinewaterquality/).

Gonads were dissected from two female and two male *A*. *planci*. Ovaries were rinsed with 1 μm filtered seawater (FSW) and placed in 10^-5^M 1-methyl adenine in FSW to induce ovulation. After approximately 60 min the eggs were collected, rinsed in FSW, checked microscopically for quality and germinal vesicle breakdown (i.e. fertile eggs). The eggs of the females were combined in a 1 L beaker. Sperm was collected from the testes and checked for motility. An equal amount of sperm from both males was combined and the number of sperm in a dilute solution was counted using a haemocytometer. Once counted, the eggs were fertilised at a sperm to egg ratio of 100:1. Fertilisation was checked and confirmed to be >90% and the eggs were rinsed in FSW to remove excess sperm.

Fertilised eggs were reared at ~27°C in two 300 L culture chambers of FSW with gentle aeration. After 24–36 h, actively swimming gastrulae were siphoned out and divided into fifty 1000 ml rearing containers (10 containers per food treatment, see below) of FSW at a density of one larvae ml^-1^. Each container had a gentle air-lift from the base to ensure mixing of the water and to maintain high levels of dissolved oxygen. The experiment was conducted in a temperature-controlled room at 27°C (±0.2°C), which was monitored using a Thermodata logger (iB-Cod Type G, Marblehead, MA, USA). FSW was changed daily over the 16-day period to settlement, with the remaining larvae monitored for a further two days. Water was changed by gentle reverse filtration with the utmost care and would not be expected to be a source of mortality. The containers were thoroughly washed and rinsed every 2–3 days to reduce biofilm accumulation. Salinity 34.5 (±0.16; n = 16), pH 8.28 (±0.007; n = 16) and DO 100.7% (±0.21; n = 16) were checked daily in replicate containers before water renewal using a Hach Hqd Portable temperature-compensated multiprobe.

### Experimental feeding treatments

Once the larvae had a complete digestive tract (~48 h post-fertilisation) they were fed daily with the tropical microalgae, *Proteomonas sulcata*, at five cell densities; 0, 100, 1000, 10000, 100000 cells ml^-1^ (n = 10 containers per treatment; Table 2). These represented chl *a* levels of 0, 0.01, 0.1, 1.0 and 10 μg chl *a* L^-1^, as determined by spectrophotometric analysis of extracted chl *a* prior to beginning the experiment, and confirmed across five random days throughout the experiment (S1 Fig.). Chlorophyll was extracted using 90% acetone and with samples kept dark and cool (~4°C) for 18–24 h before analysing by spectrophotometry [40]. Chl *a* levels were calculated using the equation: chl *a* = (11.85×A_664_)-(0.08×A_630_) to determine algal cell density [40]. The five treatments were chosen to simulate starvation/oligotrophic waters (0–100 cells ml^-1^; 0–0.01 μg chl *a* L^-1^), conditions reflecting natural background levels of nutrients on the GBR (1,000–10,000 cells ml^-1^; 0.1–1.0 μg chl *a* L^-1^), and enhanced eutrophic conditions in runoff scenarios (100,000 cells ml^-1^; 10 μg chl *a* L^-1^) (Table 1).

**Table 2:** Summary of larval rearing conditions as algal cells mL^-1^, average larvae mL^-1^, algal cells per larvae, and average mortality (see methods) on days 4, 7 and 10 (averages from n = 10; ±se).

		Algal treatment (μg chl *a* L^-1^)				
		0	0.01	0.1	1	10
**Day 4**	*Cells ml* ^*-1*^	0	100	1002	10016	100163
	*Larvae ml* ^*-1*^	0.96 (±0.06)	0.92 (±0.06)	0.98 (±0.06)	0.98 (±0.04)	1.01 (±0.05)
	*Cells larvae* ^*-1*^	0	109	1025	10249	98845
	*Mortality (%)*	4 (±4)	8.1 (±4)	2.3 (±5)	2.3 (±3)	1.3 (±4)
**Day 7**	*Cells ml* ^*-1*^	0	100	1002	10015	100147
	*Larvae ml* ^*-1*^	0.75 (±0.10)	0.66 (±0.09)	0.83 (±0.07)	0.78 (±0.11)	0.83 (±0.04)
	*Cells larvae* ^*-1*^	0	121	1211	12906	120983
	*Mortality(%)*	24.8 (±10)	33.7(±9)	17.3(±7)	22.4 (±11)	17.2(±4)
**Day 10**	*Cells ml* ^*-1*^	0	100	1000	10000	99999
	*Larvae ml* ^*-1*^	0.68 (±0.10)	0.55 (±0.07)	0.66 (±0.06)	0.66 (±0.11)	0.81 (±0.11)
	*Cells larvae* ^*-1*^	0	180	1524	15106	123186
	*Mortality (%)*	32.4(±5)	44.6(±3)	34.4(±3)	33.8(±5)	18.9(±6)

Daily water changes and renewal of food levels ensured that larval feeding did not modify the overall density of *P*. *sulcata* in each treatment throughout the experiment. This was also confirmed by spectrofluorometric assays of chl *a* levels in treatment water after 24 h and prior to water and food changes on five random days throughout the experiment (S1 Fig.). Larval densities were quantified in counts of 30 mL subsamples per treatment to ensure a consistent food ration per larvae throughout the experiment (Table 2). Mortality was determined from the number of larvae alive in daily counts. Water levels were adjusted daily to ensure that larval density remained ~1 larvae ml^-1^. The density of larvae and algae therefore remained relatively constant throughout the experiment.

### Larval development

**Fig 2:**
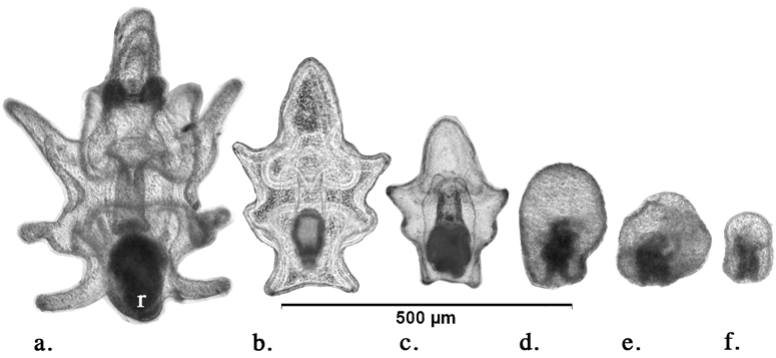
Examples of development of *Acanthaster planci* larvae. (a) Brachiolaria (day 16) with rudiment (r), (b) late-bipinnaria (day 7), (c) early-bipinnaria (day 4), (d-f) abnormal, distorted or arrested development.

Larvae were haphazardly sampled from each replicate container on day 4, 7 and 10. Approximately 50–75 ml was taken from each replicate to ensure that enough larvae were extracted for photography and analysis (20–30 larvae). The larvae were placed in 7% MgCl_2_ for ca. 15 min to relax, and then fixed in 4% paraformaldehyde in FSW. The larvae were promptly photographed to avoid post-fixation change. The first 20 larvae encountered in random samples for each replicate were photographed using a camera mounted on an Olympus DP26 microscope. The length and width of these larvae was measured using Image J software (NIH, Bethesda, MD, USA), and the larvae were scored for abnormality. Abnormal larvae had a distorted or irregular shape or were arrested at an early embryonic stage (Fig. 2). The mean of measurements from 20 larvae per replicate was used as the datum for analysis (n = 10).

### Settlement and metamorphosis

By day 16, approximately 20% of the larvae reared in the 1 μg chl *a* L^-1^ treatment had spontaneously settled across many replicates. This prompted initiation of larval settlement assays. These were done in shallow 6-well plastic culture dishes (10 mL) each with 20 competent larvae at the late brachiolaria stage with a prominent juvenile rudiment (Fig. 2a), and a fragment (~1 cm^2^) of crustose coralline algae (CCA) to induce settlement [41]. Eight replicate containers were used for each treatment (excluding the no-food treatment, which had no competent larvae). After 48 h, the percentage of newly settled juveniles was determined for each replicate (n = 8), and juveniles were photographed and their diameter measured using Image J software (as above). The sample size for juvenile measurements varied depending on the number of larvae that had metamorphosed in each treatment.

### Statistical analyses

Values for chl *a* on the GBR (determined from the eReefs resource) were analysed using a two-way ANOVA with location (Wet Tropics, Burdekin, Fitzroy) and proximity to coastline (coastal, mid-shelf, offshore) as fixed factors. Post-hoc Tukey’s HSD tests were used to determine significant differences.

Summary statistics of changes in larval length and width are presented as box and whisker plots, where the minimum, 25^th^ percentile, median, 75^th^ percentile and maximum values were calculated. Larval length, width and percent abnormal data were analysed using a one-way ANOVA for each sample day, with chl *a* levels as the fixed factor. Percent settlement and juvenile size were also analysed using one-way ANOVA with chl *a* levels as the fixed factor. All percent data were arcsine transformed, and assumptions of normality and homogeneity of variance were met, as required for ANOVA [42]. Additional two-way ANOVAs were also run with chl *a* and day as fixed factors, to determine differences across sample days. Post-hoc Tukey’s HSD tests were used to determine differences between treatments. All statistical tests were performed using JMP 9 (SAS Institute, Cary, NC, USA).

## Results

### Levels of chlorophyll a on the Great Barrier Reef

**Fig 3:**
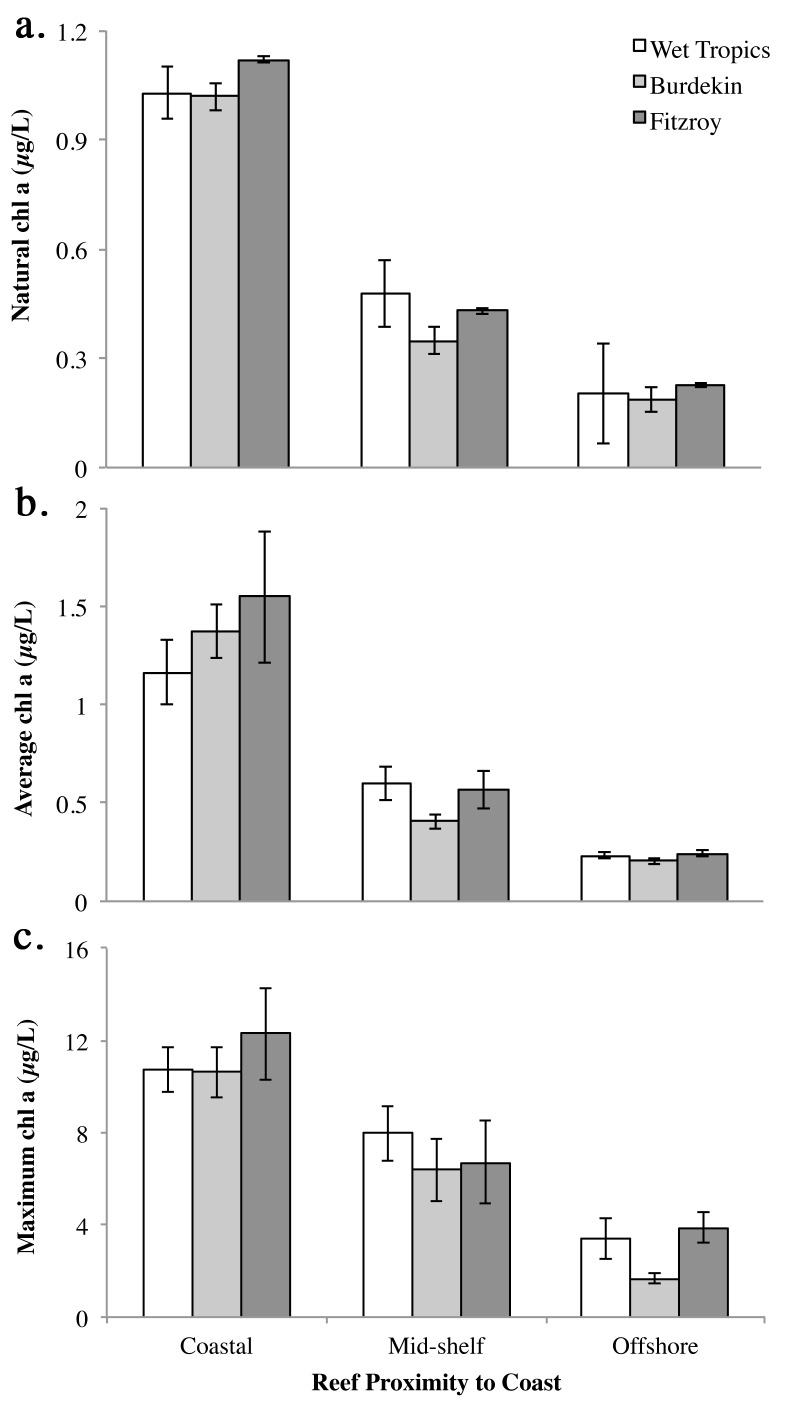
Levels of chl *a* (μg L^-1^) on the Great Barrier Reef where hotspots of *Acanthaster planci* outbreaks occur (Wet Tropics, Burdekin and Fitzroy), when larvae would be expected in the plankton (November-March). Average (a) natural chl *a* from 2011–2014 (n = 20; ±se), and (b) mean and (c) maximum chl *a* recorded for the week following major cyclone or flood events between 2009–2014 (n = 7; ±se). Data sourced from eReefs (http://www.bom.gov.au/marinewaterquality/).

Average natural levels of chl *a* on the GBR between 2011–2014 were ~1 μg chl *a* L^-1^ in coastal regions, but were lower for mid-shelf and offshore regions (~0.40 and ~0.20 μg chl *a* L^-1^, respectively) (Fig. 3; Tables 1, 3). Average levels of chl *a* for the week following major cyclone or flood events were similar to natural levels for each location (Fig. 3). However, average maximum values for the week following a major rainfall or storm event were much higher, reaching ~10 μg chl *a* L^-1^ in coastal regions, ~8 μg chl *a* L^-1^ mid-shelf, and 2–4 μg chl *a* L^-1^ offshore (Fig. 3). Peak values ranged from 5.34–23.24 μg chl *a* L^-1^ for coastal regions, to 2.13–17.00 μg chl *a* L^-1^ and 1.06–7.14 μg chl *a* L^-1^ on mid-shelf and offshore regions, respectively (Table 1). There was no significant difference in chl *a* at different latitudinal locations (Wet Tropics, Burdekin, Fitzroy). A Tukey’s HSD test revealed that chl *a* levels were significantly higher in coastal waters both naturally and following flood, rainfall or cyclone events (Fig. 3; Table 3).

**Table 3:** Two-way ANOVA of average natural levels of chl *a* where hotspots of *Acanthaster planci* outbreaks occur on the Great Barrier Reef, for when larvae would be expected in the plankton (November-March) from 2011–2014 (n = 20), and mean and maximum chl *a* values recorded for the week following seven major events (November-March, 2009–2014; n = 7).

	Factor	df	*F*-ratio	*p*-value	Tukey’s HSD
**Natural chl *a***	*Location*	2	1.02	0.36	-
	*Proximity*	2	141.3	< 0.0001	Coast > Mid-shelf > Offshore
	*Interaction*	4	0.40	0.81	-
	*Error*	171			
	*Total*	179			
**Event Mean chl *a***	*Location*	2	1.09	0.34	-
	*Proximity*	2	33.0	< 0.0001	Coast > Mid-shelf > Offshore
	*Interaction*	4	0.40	0.81	-
	*Error*	54			
	*Total*	62			
**Event Maximumchl *a***	*Location*	2	0.77	0.47	-
	*Proximity*	2	52.9	< 0.0001	Coast > Mid-shelf > Offshore
	*Interaction*	4	0.83	0.51	-
	*Error*	54			
	*Total*	62			

Data sourced from eReefs (http://www.bom.gov.au/marinewaterquality/). Location refers to outbreak hotspots Wet Tropics (Cairns/Lizard Island), Burdekin (Townsville) and Fitzroy (Swains Reef); Proximity is distance from mainland (coastal, mid-shelf, offshore).

### Larval growth and survival

**Fig 4:**
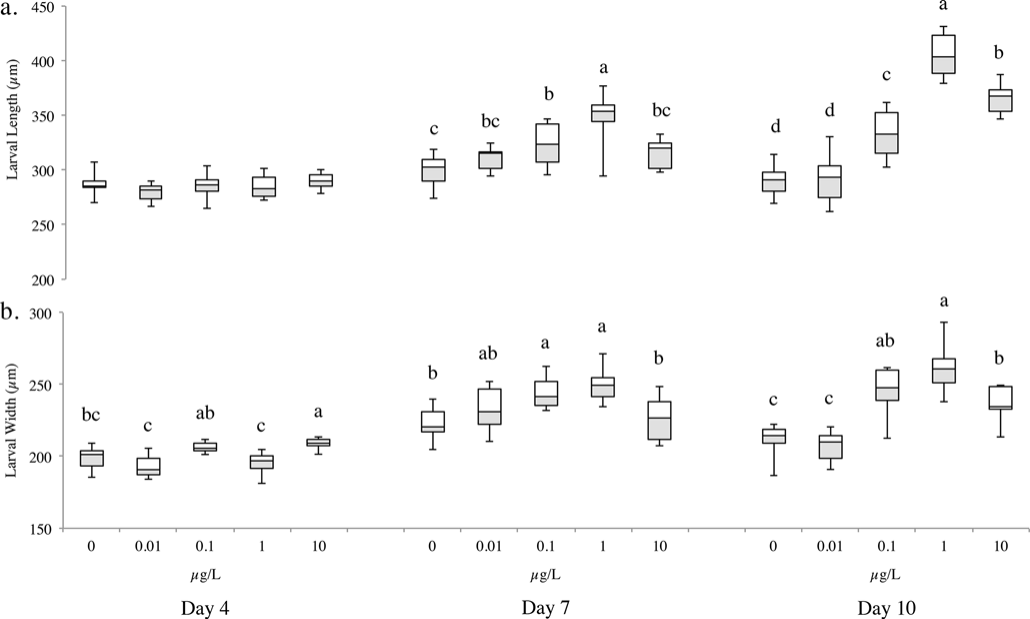
Larval (a) length and (b) width for *Acanthaster planci* reared in five algal concentrations, represented as chl *a* concentration (μg L^-1^) on days 4, 7 and 10 (n = 10). Boxes represent the interquartile range (25 and 75^th^ percentile), the horizontal line is the median, and the whiskers represent the data range. Tukey’s HSD test: levels not connected by the same letter are significantly different (within each day).

There was a significant effect of algal concentration on larval length (F_(8,133)_ = 26.4; p <0.0001) and width (F_(8,133)_ = 12.6; p <0.0001). At day 4, there was no significant difference in length (F_(4,45)_ = 1.5; p = 0.21), but there was for width (F_(4,45)_ = 12.5; p <0.0001). There was little larval growth in the no and low food treatments (0 and 0.01 μg chl *a* L^-1^) (Fig. 4). On days 7 and 10, the largest larvae were found in the 1 μg chl *a* L^-1^ food treatment (Day 7: length: F_(4,44)_ = 12.9; p <0.0001; width: F_(4,44)_ = 8.1; p <0.0001; Day 10: length: F_(4,44)_ = 67.9; p <0.0001; width: F_(4,44)_ = 29.8; p <0.0001) (Fig. 4). These larvae were 7–16% longer and 1–11% wider than all other treatments by day 7, and 9–28% longer and 6–21% wider by day 10.

**Fig 5:**
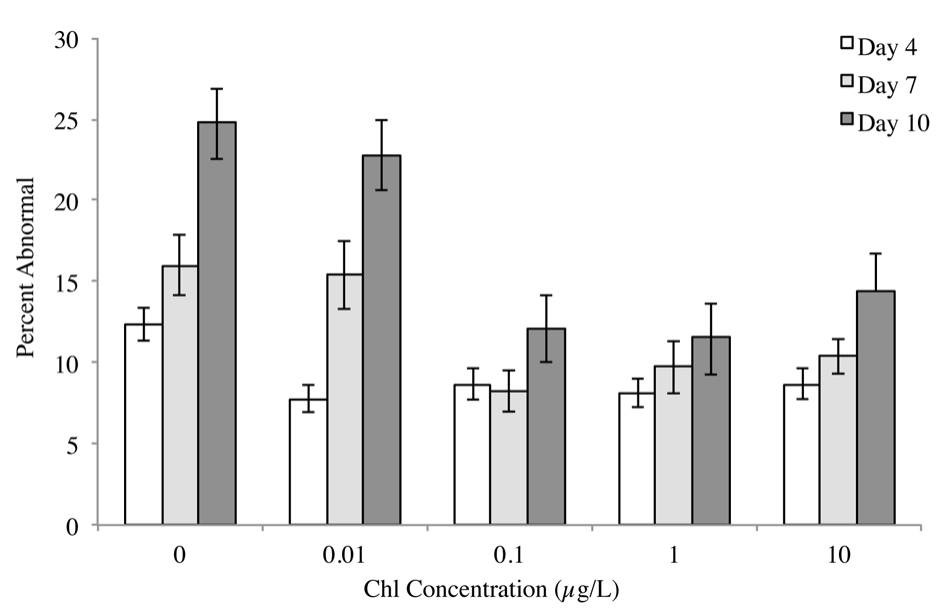
Average percent abnormality (±se) of *Acanthaster planci* reared in five algal concentrations, represented as chl *a* concentration (μg L^-1^) on days 4, 7 and 10 (n = 10).

Abnormality was highest for larvae in the no food treatment at day 7 (16%) and day 10 (25%) (Fig. 5). On day 7, the percentage of normal larva was highest in the background chl treatment (0.1 μg chl *a* L^-1^) (F_(4,44)_ = 4.6, p = 0.0036; Tukey’s HSD: 0.1 ≥ 1 = 10 ≥ 0.01 = 0 μg chl *a* L^-1^). By day 10, the percentage of normal larva was highest in the 0.1 and 1 μg chl *a* L^-1^ treatments (F_(4,44)_ = 8.4, p <0.0001; Tukey’s HSD: 1 = 0.1 ≥ 10 ≥ 0.01 ≥ 0 μg chl *a* L^-1^) (Fig. 5).

There was a significant increase in mortality across the sampling days (F_(2,135)_ = p <0.0001; Tukey’s HSD: day 4 > day 7 = day 10;). By day 10, survivorship was lowest (45%) in the lowest food treatment (0.01 μg chl *a* L^-1^), and was highest (19%) in the high-food treatment (10 μg L^-1^) (Table 1). While there was a clear trend in larval survival with respect to food treatment over time, there was no significant effect of feeding treatment on mortality within each sampling day.

### Settlement and metamorphosis

**Fig 6:**
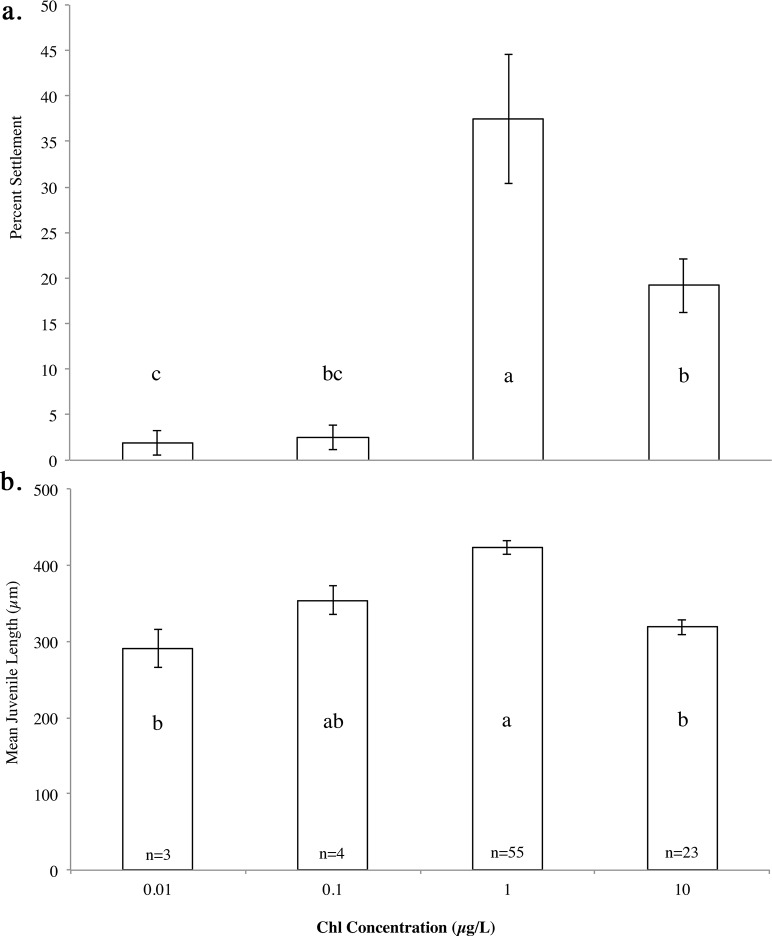
Average (a) percent settlement and (b) size of recently settled *Acanthaster planci* juveniles at day 18, following rearing larval rearing in five algal concentrations represented as chl *a* concentration (μg L^-1^) across all settlement assays (±se). Tukey’s HSD test: levels not connected by the same letter are significantly different.

There was a significant difference in settlement success (F_(3,26)_ = 17.7, p <0.0001) and newly settled juvenile size (F_(3,81)_ = 18.6, p <0.0001) depending on the larval rearing conditions. A Tukey’s HSD test revealed that larvae fed at 1 μg chl *a* L^-1^ had the highest settlement success 37.5% (Fig. 6a) and the largest juveniles (Fig. 6b). Settlement success for competent larvae reared at 10 μg chl *a* L^-1^ was ~20% (Fig. 6a), with smaller juveniles (Fig. 6b). Settlement in the two low-food treatments (0.1 and 0.01 μg chl *a* L^-1^) was ~2%, with the smallest juveniles observed at 0.01 μg chl *a* L^-1^.

## Discussion

To address our hypotheses on the response of *A*. *planci* larvae to food conditions ranging from starvation to satiation, larvae were reared under carefully controlled conditions with respect to algal density, chl *a* levels and food ration per larva. As typical of the planktotrophic larvae of other asteroids and marine invertebrates in general [30,43–48], food levels had a strong influence on larval growth to the metamorphic stage in *A*. *planci*, with faster development to the juvenile with increased food ratio, up to a limit. We avoided the confounding influence of increasing food ration per larva due to mortality; a consideration rarely incorporated into larval culture studies (but see: [43]). In previous studies of *A*. *planci*, larval:algal cell ratios varied (e.g. [14,30,37]), and so it is not possible to understand the absolute level of food available to individual larvae in experimental treatments.

A wealth of studies on the planktotrophic larvae of asteroids and other echinoderms show the importance of food levels on larval success [22,47–52]. Food limitation affects larval size, pelagic larval duration and post-metamorphic success [48–50,52]. As a tropical species, the larvae of *A*. *planci* are suggested to be resilient to naturally oligotrophic conditions [21,22]. However, we found that larvae in no and low algal food treatments (0–100 cells ml^-1^) were the smallest and also exhibited high abnormality and mortality, as reported previously for *A*. *planci* [30,37]. Unfed larvae did not develop beyond the early bipinnaria stage when the experiment ended at 18 days. Thus, counter to the *larval resilience hypothesis*, which posits that *A*. *planci* larvae are resilient to low phytoplankton levels ultilising alternate sources of nutrients (e.g. DOM, bacteria) [21], our data shows that low food levels (0–0.01 μg chl *a* L^-1^) are unlikely to sustain larvae (but see: [53]). However, it is not known whether surviving larvae reared in these oligotrophic levels may have developed to a more advanced stage if reared beyond 18 days, or whether they could recover if provided with an event-driven pulse of food, as shown for other asteroid and invertebrate larvae [49,54,55].

It has been suggested that larvae reared at ≤0.8 μg chl *a* L^-1^ are food limited, based on laboratory cultures where chl *a* levels were extrapolated from published values [14,37]. Within the feeding regimes used here per larva, *A*. *planci* larvae reared at 1 μg chl *a* L^-1^ were largest and exhibited spontaneous settlement after 16 days. This is lower than the ~2–6.5 μg chl *a* L^-1^ optimal value suggested in previous studies [14,30,37]. The highest phytoplankton level used (10 μg chl *a* L^-1^) was deleterious to larval development. Our food treatments did not incorporate the developmental trigger value of 0.8 μg chl *a* L^-1^ modeled to be an important cut off for the success of *A*. *planci* larvae [14], a food level that warrants further examination.

Inshore and mid-shelf peaks in chl *a* concentrations on the GBR, and consequent increases in zooplankton abundance, are directly related to physical factors such as river discharge and summer upwelling events [38,56]. The larvae of *A*. *planci* are likely to utilize a diversity of algal species, including the nano- (2–10 μm) and picoplankton (<2 μm) known to be abundant in tropical waters [38]. However these small algae exhibit little seasonal variation and shift to larger phytoplankton species (>10 μm) in summer in response to upwelling or river runoff events [38,39]. Increased abundance of larger phytoplankton species in summer may benefit the larvae of *A*. *planci*, which are known to effectively feed on particles between 6 and 20 μm [57], a size range that includes *Proteomonas sulcata* (~10 μm), the species used here.

The optimal food level here (1 μg chl *a* L^-1^) is often recorded on the GBR as a natural background condition along the coast, and as peak eutrophic conditions on mid-shelf and offshore reefs following storm events when the larvae would be expected to be in the plankton (Table 1, S1 Table) [28,35,38,39,58,59]. On the GBR, the frequency and intensity of outbreaks are especially high for mid-shelf reefs [6,60], rather than offshore or coastal reefs [12]. With respect to mean levels of surface productivity, it seems that mid-shelf regions on the GBR have a high background level of phytoplankton (0.4–0.6 μg chl *a* L^-1^; Table 1, S1 Table), which may support successful development of *A*. *planci* larvae irrespective of flood or storm events. Based on the response of larvae to the high food treatment (10 μg chl *a* L^-1^), peak values of eutrophication recorded for mid-shelf and coastal regions on the GBR (~8 and ~12 μg chl *a* L^-1^, respectively) may create suboptimal conditions for *A*. *planci* larvae, albeit in short-lived pulses following storm events.

Linking individual flood events to *A*. *planci* outbreaks is tenuous considering the delay time from larval development, recruitment, juvenile development and the emergence or detection of adult populations (>3 years) [12], although laboratory studies indicate this could be shorter (~18–21 months) [61]. Population connectivity and recruitment success are modelled to influence patterns of *A*. *planci* outbreaks [15], and larval experience can have significant carry-over effects post-settlement [27,48]. *Acanthaster planci* larvae have a pelagic larval duration ranging between 9–42 days [10,27]. Here, larvae reared at 1 μg chl *a* L^-1^ spontaneously settled in 16 days, with the greatest settlement success and juvenile size (a measure of recruit quality). Thus, larval experience of *A*. *planci* impacts juvenile quality, as shown for other asteroids, echinoids, gastropods and barnacles [49,54,55,62]. As found for *A*. *planci*, well-fed asteroid and echinoid larvae develop faster, have a shorter pelagic larval duration and have enhanced post-metamorphic success [49,62]. The settlement and metamorphic stages are likely bottlenecks for the overall success of *A*. *planci*, and our results—through to settlement—indicate increased success to the juvenile stage in the two higher food treatments.

Converse to the suggestion that *A*. *planci* larvae are particular about their settlement substratum [27,41,63], spontaneous settlement was observed in our plastic culture chambers for larvae fed at 1 μg chl *a* L^-1^. This shows that the larvae do not require a coralline algal cue *per se* for settlement. It is likely that the larvae were responding to a biofilm on the containers, even though the containers were regularly cleaned. In addition, when settling to CCA, which is a common cue for settlement in marine larvae [64], it is most likely a biofilm that they are responding to [41]. However, overall settlement was low, as noted for *A*. *planci* in other studies [27,63], where predation of metamorphosing larvae by fauna in the CCA matrix was suggested to be a major factor [63]. Although previous studies report 100% survival of *A*. *planci* larvae reared in chl *a* levels extrapolated to be ~2–5 μg chl *a* L^-1^ [14,37], overall survival included abnormal and regressed larvae, an assessment criterion not comparable with the more typical determination of larval success across developmental stages [47,49,65], as in the present study.

With global change projections of increased cyclone and storm events over the coming decades, it seems that the optimal chl *a* levels for the larvae of *A*. *planci* will be reached more frequently on the GBR and other tropical reefs. Future increases in runoff conditions and summer upwelling may continue to enhance larval success and the potential for *A*. *planci* outbreaks. A caveat for *A*. *planci* in this scenario is that increased nutrient loading to marine waters and consequent microbial respiration accelerates seawater acidification [66], a factor deleterious to development in *A*. *planci* [67,68]. At projected near future levels of ocean warming and acidification, *A*. *planci* larvae may exhibit impaired development and lower settlement success [67,68]. Thus, it is likely that there will be complex and somewhat unpredictable interplay between the success of *A*. *planci* larvae and its calcifying coral prey in a future ocean.

## Supporting Information

S1 Table Range of values of natural chl *a* concentrations (μg L^-1^), determined from discrete water samples taken across the Great Barrier Reef (data from [39]). Note: values are congruent with natural mean levels of chl *a* calculated from eReefs (see: Table 1).
(DOCX)

S1 Fig Average chl *a* concentration (μg chl *a* L^-1^) in treatment across five random sample days, at the initial feeding concentration and 24 hr following larval feeding (n = 5; ±se). There was no significant difference in algal concentration between the initial and final concentration (two-way ANOVA: F_(4,49)_ = 0.02; p = 0.99).
(TIF)
