# Supplementary material for: Larval Starvation to Satiation: Influence of Nutrient Regime on the Success of Acanthaster planci
Source: PLoS One. 2015 Mar 19;10(3):e0122010. doi: 10.1371/journal.pone.0122010 (PMC4366153; doi:10.1371/journal.pone.0122010)
Supplement: S1 Table — Note: values are congruent with natural mean levels of chl a calculated from eReefs (see: Table 1). (DOCX) [file pone.0122010.s001.docx]

**Table S1: Range of values of natural chl *a* concentrations (µg L^-1^)**, determined from discrete water samples taken across the Great Barrier Reef (data from [39]). Note: values are congruent with natural mean levels of chl *a* calculated from eReefs (see: Table 1).

| **Region** |  | **Chl *a* (µg L^-1^)** |
| --- | --- | --- |
| Far Northern GBR  (*Wet Tropics*) | *Inner* | 0.11-0.39 |
|  | *Outer* | 0.12-0.41 |
| Cairns | *Inner* | 0.11-1.08 |
|  | *Outer* | 0.07-0.37 |
| Townsville  (*Burdekin*) | *Inner* | 0.18-1.17 |
|  | *Outer* | 0.05-0.32 |
| Whitsundays | *Inner* | 0.10-0.63 |
|  | *Outer* | 0.06-0.26 |
| Capricorn Bunker  (*Fitzroy*) | *Inner* | 0.16-1.30 |
|  | *Outer* | 0.17-1.10 |
