# Supplementary figures and images for: Larval Starvation to Satiation: Influence of Nutrient Regime on the Success of Acanthaster planci
Source: PLoS One. 2015 Mar 19;10(3):e0122010. doi: 10.1371/journal.pone.0122010 (PMC4366153; doi:10.1371/journal.pone.0122010)

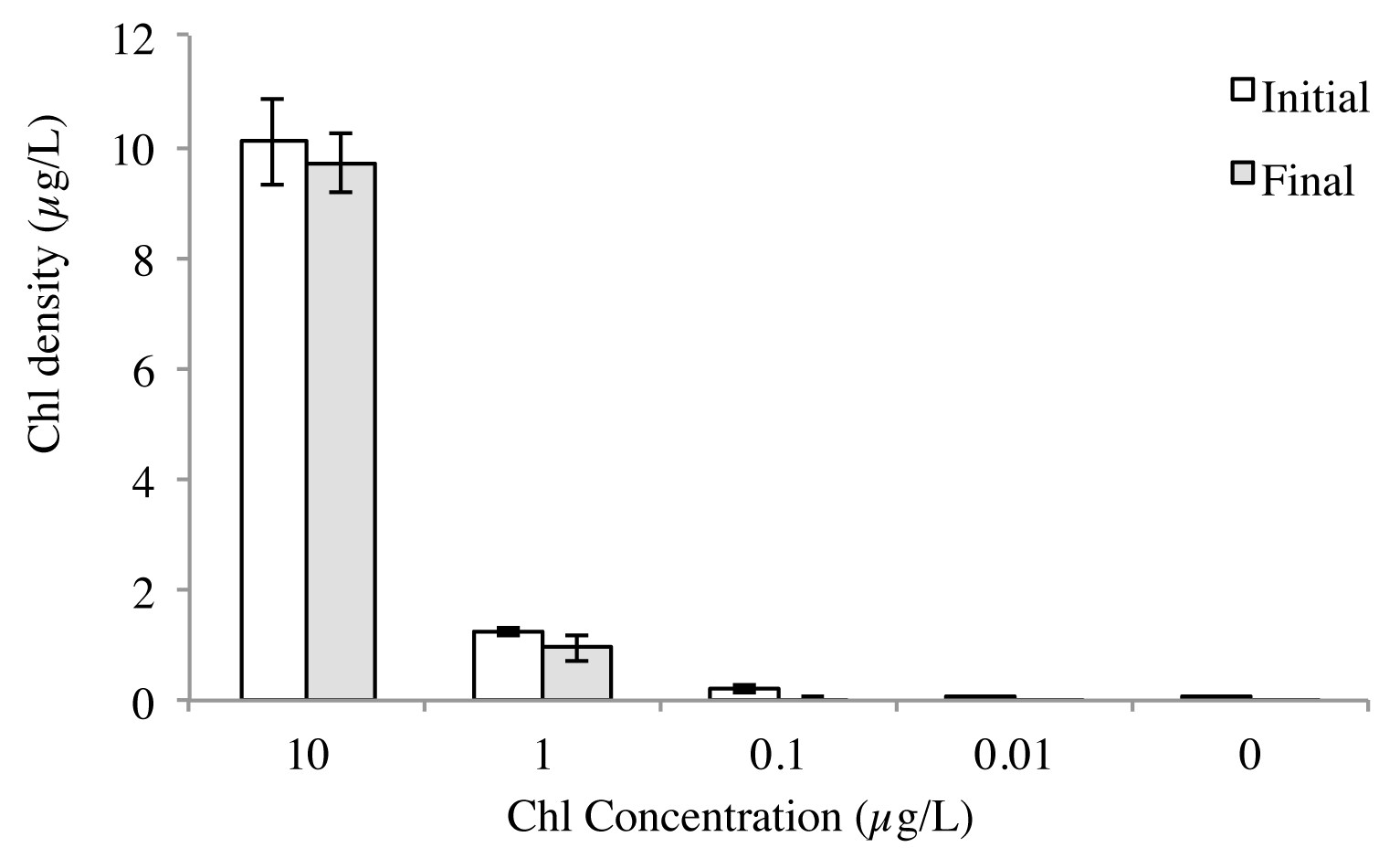

Supplement: S1 Fig — There was no significant difference in algal concentration between the initial and final concentration (two-way ANOVA: F(4,49) = 0.02; p = 0.99). (TIF) [file pone.0122010.s002.tif]
